# Hyperspectral Imaging for Early Detection and Severity Grading of Potato Bacterial Wilt

**DOI:** 10.3390/plants15111706

**Published:** 2026-05-31

**Authors:** Zhuo Chen, Zhendong Lan, Xi-Ou Xiao, Xi Zhu, Yu Zhang, Xidan Pang, Hui Jin

**Affiliations:** 1 State Key Laboratory of Tropical Crop Breeding, Sanya Research Institute, Chinese Academy of Tropical Agricultural Sciences, Sanya 572024, China; chenzhuo@catas.cn (Z.C.); 2 Key Laboratory of Tropical Fruit Biology, Ministry of Agriculture and Rural Affairs/Key Laboratory of Postharvest Physiology and Technology of Tropical Horticultural Products of Hainan Province, South Subtropical Crops Research Institute, Chinese Academy of Tropical Agricultural Sciences, Zhanjiang 524091, China; 3 College of Horticulture and Forestry Science, Huazhong Agricultural University, Wuhan 430070, China

**Keywords:** *Ralstonia solanacearum*, bacterial wilt, hyperspectral imaging, early disease detection, precision agriculture

## Abstract

Potato (*Solanum tuberosum*) is a vital global non-cereal food crop severely threatened by bacterial wilt, caused by *Ralstonia solanacearum*(*R*. *solanacearum*). Conventional diagnostics like PCR and ELISA, though effective, are destructive and time-consuming, limiting large-scale field applications. This study investigates hyperspectral imaging (HSI) as a non-invasive, rapid, and accurate alternative for early detection and severity grading of potato bacterial wilt. Using a portable HSI system (400–1000 nm), spectral data were collected from inoculated potato plants (‘Longshu No. 7’) at 0, 24, 48, and 72 h post-inoculation, alongside disease severity assessment (grades 0–4). After comprehensive spectral preprocessing and feature band extraction via Competitivse Adaptive Reweighted Sampling (CARS), we developed two distinct sets of models: one for early detection (temporal classification) using Partial Least Squares-Discriminant Analysis (PLS-DA) and Principal Component Analysis-Linear Discriminant Analysis (PCA-LDA), and another for severity grading. The SNV + SG + MC + PLS-DA model achieved exceptional accuracy, exceeding 97% for early detection, while the MSC + SG + MC + CARS + PLS-DA model yielded >97% accuracy for severity grading. These results were supported by low misclassification rates in confusion matrices. This work establishes a robust HSI-based framework for high-throughput screening of resistant potato germplasm and advances precision agriculture strategies for bacterial wilt management.

## 1. Introduction

Potato (*Solanum tuberosum*) is the most widely consumed non-cereal food crop globally [1,2]. Bacterial wilt, caused by *R*. *solanacearum*, poses a significant threat to potato production, with annual economic losses estimated at $950 million [3,4]. This soil-borne bacterium invades the plant’s vascular system, leading to wilting and plant death [5,6]. Critically, during early infection stages, plants often lack visible symptoms, rendering visual inspection ineffective [7]. Traditional detection methods, such as enzyme-linked immunosorbent assay (ELISA) and polymerase chain reaction (PCR), are accurate but require destructive sampling, specialized expertise, and are time-consuming, making them unsuitable for large-scale, in-field monitoring [8]. Consequently, there is a pressing need for rapid, non-destructive diagnostic technologies.

Given the limitations of conventional diagnostics, hyperspectral imaging (HSI) has emerged as a promising non-destructive tool for precision agriculture [9,10]. Its application spans various plant diseases, including late blight in potatoes [11] and bacterial wilt in bananas [12]. Beyond traditional machine learning algorithms such as PLS-DA and PCA-LDA, deep learning approaches—including convolutional neural networks (CNNs)—have recently shown potential for extracting spatial–spectral features from hyperspectral data [13,14]. However, deep learning typically requires large annotated datasets and substantial computational resources, which may not be available in breeding or resource-limited settings [15,16]. Therefore, this study employs traditional machine learning methods, which are well suited for moderate-sized datasets and provide interpretable models. In the context of potato bacterial wilt, systematic investigations using HSI for early, pre-symptomatic detection and severity grading remain limited, underscoring the need for the present study.

Current resistance assessment for bacterial wilt in potatoes often relies on manual disease scoring, which is subjective and prone to human error [17]. HSI offers an objective alternative by quantifying spectral changes associated with disease progression. Recent advancements in sensor technology and data analytics, including resource-efficient federated learning for disease classification and cloud/fog computing architectures for smart agriculture, underscore the potential for scalable, automated disease monitoring systems [18,19].

This study aims to develop and validate HSI-based models specifically for two objectives: (1) early detection of *R. solanacearum* infection in potatoes before symptom visibility, and (2) accurate severity grading of bacterial wilt. We employed a portable HSI system within the 400–1000 nm range, a common and effective spectrum for assessing plant pigments and water content. We systematically evaluated multiple spectral preprocessing techniques and feature selection methods (CARS) to optimize the performance of PLS-DA and PCA-LDA models. Our work establishes a foundation for a non-invasive, high-throughput system for potato bacterial wilt management, contributing to the broader goals of Society 5.0 in agriculture by integrating sensing technology with data-driven decision-making.

## 2. Results

### 2.1. Canopy Hyperspectral Response Characteristics

**Figure 1:**
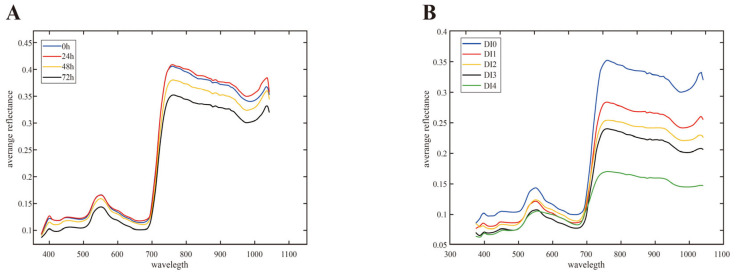
Canopy spectral reflectance. ((**A**): canopy spectral at different stages after *R. solanacearum* inoculation; (**B**): canopy spectral reflectance of different disease severity of potato after inoculation with *R. solanacearum*).

Spectral reflectance changes were observed post-inoculation (Figure 1A). In the visible range (400–700 nm), differences between 0 h and 24 h were minimal, suggesting no initial chlorophyll degradation. In the near-infrared (NIR, 700–1000 nm), reflectance at 24 hpi was higher than at 0 hpi, potentially indicating early changes in leaf internal structure or water content before visible wilting. By 48 h and 72 hpi, reflectance decreased significantly across the spectrum, consistent with disease progression.

For disease severity, a clear gradient in reflectance was observed: healthy plants (grade 0) had the highest reflectance, which progressively decreased with increasing disease severity (Grade 1 > 2 > 3 > 4) across the entire spectrum (Figure 1B). The difference was most pronounced in the NIR region, highlighting its sensitivity to structural damage and water loss. These observed spectral differences motivated the application of preprocessing and classification techniques to quantitatively distinguish infection stages and severity levels.

### 2.2. Data Preprocessing

**Figure 2:**
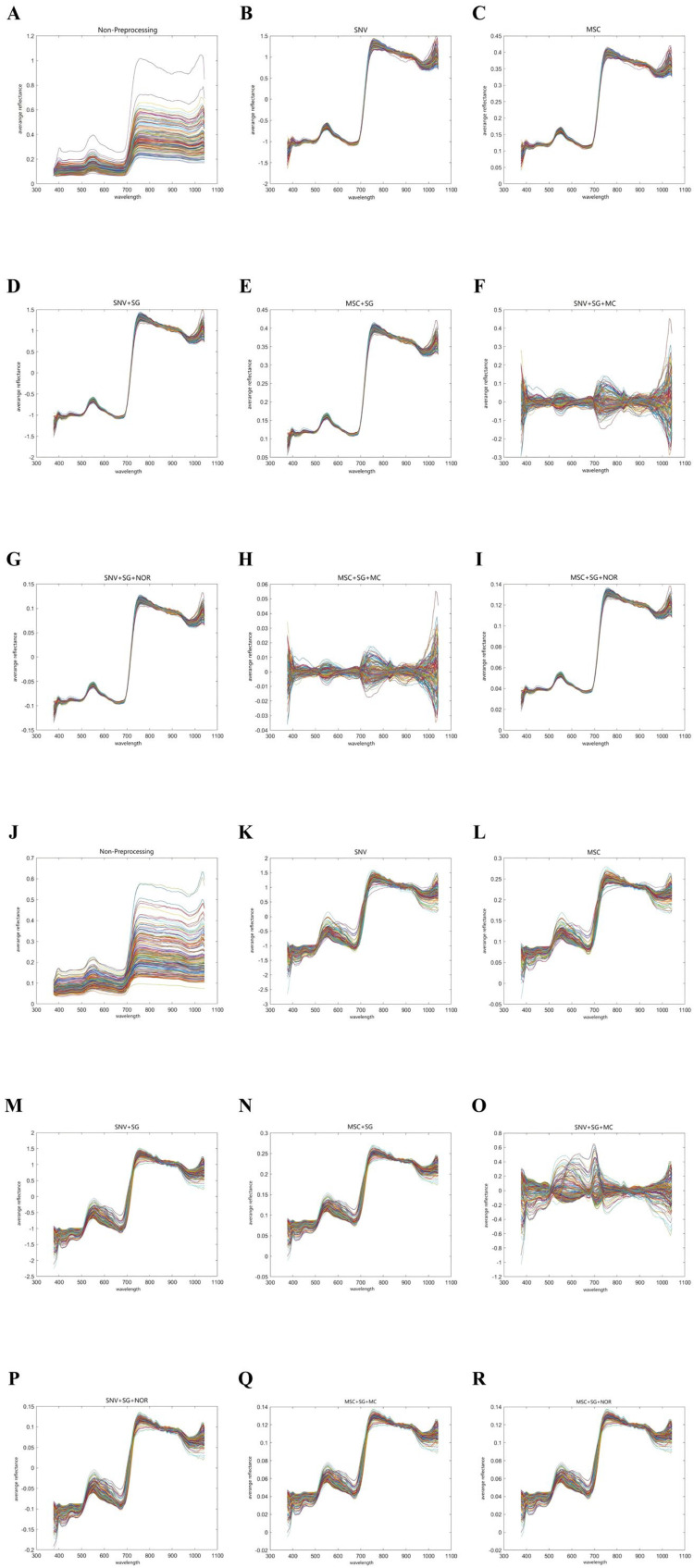
Data preprocessing. (The colored lines represent different stray light, noise, and baseline drift, respectively. (**A**–**I**): the pretreatment of the spectral data following the potato inoculation with *R. solanacearum* at 0 h, 24 h, 48 h, and 72 h; (**J**–**R**): pretreatment of canopy spectral reflectance for different severity of potato bacterial wilt after inoculation with *R. solanacearum*).

Preprocessing effectively reduced noise and baseline effects (Figure 2). Standard Normal Variate (SNV) and Multiplicative Scatter Correction (MSC) were particularly effective in mitigating scattering effects, resulting in more concentrated and smooth spectral profiles. To systematically evaluate the impact of preprocessing on classification performance, we subsequently developed PLS-DA and PCA-LDA models using the preprocessed data.

### 2.3. PLS-DA Modeling Analysis

Early Detection: The PLS-DA model performance for early detection is summarized in Table 1. The unprocessed data yielded 87% accuracy for both training and test sets. Preprocessing combinations like SNV + SG + MC and MSC + SG + MC significantly enhanced performance, achieving training accuracies >98% and test accuracies of 97% and 94%, respectively. The SNV + SG + MC + PLS-DA model demonstrated superior and robust performance, with most sensitivity and specificity values at 100%, effectively distinguishing uninoculated (0 h) from inoculated samples (24 h, 48 h, 72 h). While PLS-DA showed robust performance, we also evaluated PCA-LDA as an alternative dimensionality reduction approach to compare methodological efficacy. The associated confusion matrix (Table 1) showed minimal misclassification.

Severity Grading: For severity grading, preprocessing was crucial (Table 1). Models like MSC + SG + MC + PLS-DA and MSC + SG + NOR + PLS-DA achieved test accuracies exceeding 80%, a substantial improvement over the unprocessed data. Detection of healthy plants (level 0) consistently showed the highest sensitivity and specificity across all models.

### 2.4. PCA-LDA Modeling Analysis

Early Detection: PCA-LDA model results are shown in Table 2. Performance was more variable and generally lower than PLS-DA. The MSC + SG + MC + PCA-LDA combination yielded the best results, with 95% (training) and 85% (test) accuracy. In contrast, MSC and MSC + SG preprocessing alone led to poor performance (25% test accuracy), highlighting the sensitivity of PCA-LDA to optimal preprocessing.

Severity Grading: For severity grading, the MSC + SG + MC + PCA-LDA model again performed best, achieving 85.00% (training) and 82.00% (test) accuracy, compared to 70% for unprocessed data.

### 2.5. CARS Feature Band Extraction

The Competitive Adaptive Reweighted Sampling (CARS) method was employed to select informative wavelengths, reducing data dimensionality. The CARS parameters were set as follows: Monte Carlo sampling runs: 50; Threshold (α): 0.05.

### 2.6. CARS Feature Extraction and PLS-DA Discrimination Analysis

Early Detection: Combining CARS with PLS-DA further optimized the models (Table 3). The MSC + SG + MC + CARS + PLS-DA and SNV + SG + CARS + PLS-DA models achieved test accuracies of 97% and 100%, respectively, confirming the utility of feature selection for building parsimonious and highly accurate early detection models.

Severity Grading: For severity grading, the SNV + SG + NOR + CARS + PLS-DA model performed best, with 87% (training) and 89% (test) accuracy (Table 3).

### 2.7. CARS Feature Extraction and PCA-LDA Discriminant Analysis

Early Detection: When CARS-extracted features were used with PCA-LDA (Table 4), SNV + SG + CARS + PCA-LDA and MSC + SG + MC + CARS + PCA-LDA showed the best predictive performance for early detection, with test accuracies of 90%.

Severity Grading: For severity grading, SNV + SG + MC + CARS + PCA-LDA, SNV + SG + NOR + CARS + PCA-LDA, and MSC + SG + MC + CARS + PCA-LDA achieved robust performance, with training and test accuracies around 84%, and sensitivity/specificity for each class > 60% (Table 4). To provide an overview of the relative performance of all models, a comprehensive comparison is presented in Section 2.8.

### 2.8. Comprehensive Comparison of Data Processing Methods and Model Performance

The performance of various preprocessing techniques and statistical models, including PLS-DA and PCA-LDA, was evaluated for their effectiveness in early detection and severity grading of potato bacterial wilt. This comprehensive analysis highlights the strengths and limitations of different methods.

#### 2.8.1. Model Accuracy and Sensitivity

The highest accuracy was achieved using the PLS-DA model combined with SNV + SG + MC preprocessing, with accuracy rates exceeding 97% for early detection and severity grading. This combination proved particularly effective in distinguishing healthy samples from those infected at different time points. In comparison, PCA-LDA models showed slightly lower accuracy, with the best results (MSC + SG + MC preprocessing) achieving 90% for early detection and 82% for severity grading (Table 5 and Table 6).

#### 2.8.2. Robustness Across Preprocessing Techniques

PLS-DA models demonstrated remarkable robustness across different preprocessing techniques, maintaining high accuracy even with simpler combinations like SNV + SG. On the other hand, PCA-LDA models were more sensitive to preprocessing variations. For instance, accuracy dropped significantly to 20% with MSC preprocessing alone, highlighting its dependence on optimal preprocessing combinations.

#### 2.8.3. Computational Efficiency

In terms of computational efficiency, PCA-LDA models outperformed PLS-DA due to their simpler framework, making them suitable for rapid, on-site analysis. However, for applications requiring high precision and reliability, such as breeding program evaluations, PLS-DA models provided better overall performance despite their higher computational demands.

#### 2.8.4. Feature Extraction with CARS

Feature extraction using the Competitive Adaptive Reweighted Sampling (CARS) method significantly improved model performance by reducing data complexity while retaining key spectral information. PLS-DA models benefited the most from this approach, with SNV + SG + CARS and MSC + SG + CARS preprocessing achieving nearly perfect test accuracy (97% and 100%, respectively). In comparison, PCA-LDA models saw moderate improvements, achieving maximum accuracy rates of 90% for test datasets with SNV + SG + CARS preprocessing.

#### 2.8.5. Sensitivity to Disease Severity Levels

For severity grading, both models showed a progressive decrease in reflectance accuracy as disease severity increased, reflecting structural changes in plant tissues. PLS-DA models were more effective in detecting subtle differences in reflectance, providing higher sensitivity and specificity for each severity level compared to PCA-LDA.

#### 2.8.6. Overall Performance Evaluation

The SNV + SG + MC + PLS-DA model emerged as the most accurate and robust approach for both early detection and severity grading. Its ability to handle complex spectral data and maintain high precision makes it ideal for high-throughput applications, such as germplasm screening. Meanwhile, PCA-LDA with MSC + SG + MC preprocessing offers a computationally efficient alternative for scenarios requiring rapid, preliminary assessments.

## 3. Discussion

This study successfully demonstrates the potential of HSI for the non-invasive early detection and severity grading of potato bacterial wilt. Our key innovation lies in the development of two specialized, high-accuracy models using a portable HSI system under controlled conditions. To further strengthen the biological relevance of HSI-based severity grading, it is essential to correlate spectral data with direct measurements of pathogen colonization. Quantitative PCR and bacterial enumeration have been widely used as reference standards in plant pathology [20]. In future experiments, collecting tissue samples from the same plants used for HSI acquisition and quantifying bacterial load would enable a direct calibration of spectral indices against actual disease intensity. Such integration would not only validate the HSI models but also provide insights into the physiological basis of spectral changes, such as the relationship between water content shifts and xylem colonization by *R. solanacearum*.

### 3.1. Early Detection of Potato Bacterial Wilt Based on Hyperspectral Data

The ability to detect infections as early as 24 h post-inoculation, before visible symptoms appear, is a significant advancement over traditional methods. The spectral changes in the NIR region at 24 hpi (Figure 1A) likely correspond to alterations in leaf water potential and cellular structure caused by the initial vascular colonization by *R. solanacearum*, as the pathogen begins to impede water transport [20]. This aligns with findings in other crops where pre-symptomatic detection was achieved [21,22].

The superior performance of the SNV + SG + MC + PLS-DA model can be attributed to PLS-DA’s strength in handling multicollinear spectral data by finding latent variables that maximize covariance with the class labels. In contrast, PCA-LDA, which first reduces dimensions in an unsupervised manner (PCA) before classification (LDA), may discard biologically relevant variance, making it more sensitive to preprocessing and less robust for this specific task. The high accuracies (>97%) achieved, corroborated by low misclassification in confusion matrices, underscore the model’s reliability. Building on the successful early detection models, we next evaluated whether HSI could also differentiate among distinct disease severity levels. While high accuracy can raise concerns about overfitting, the use of an independent test set and Venetian blinds cross-validation provides a rigorous assessment. Nevertheless, future validation with larger, independent datasets from different genotypes and environments is recommended to confirm generalizability, a common practice in robust model development as seen in studies on smart agriculture and cloud-based analytics.

**Figure 3:**
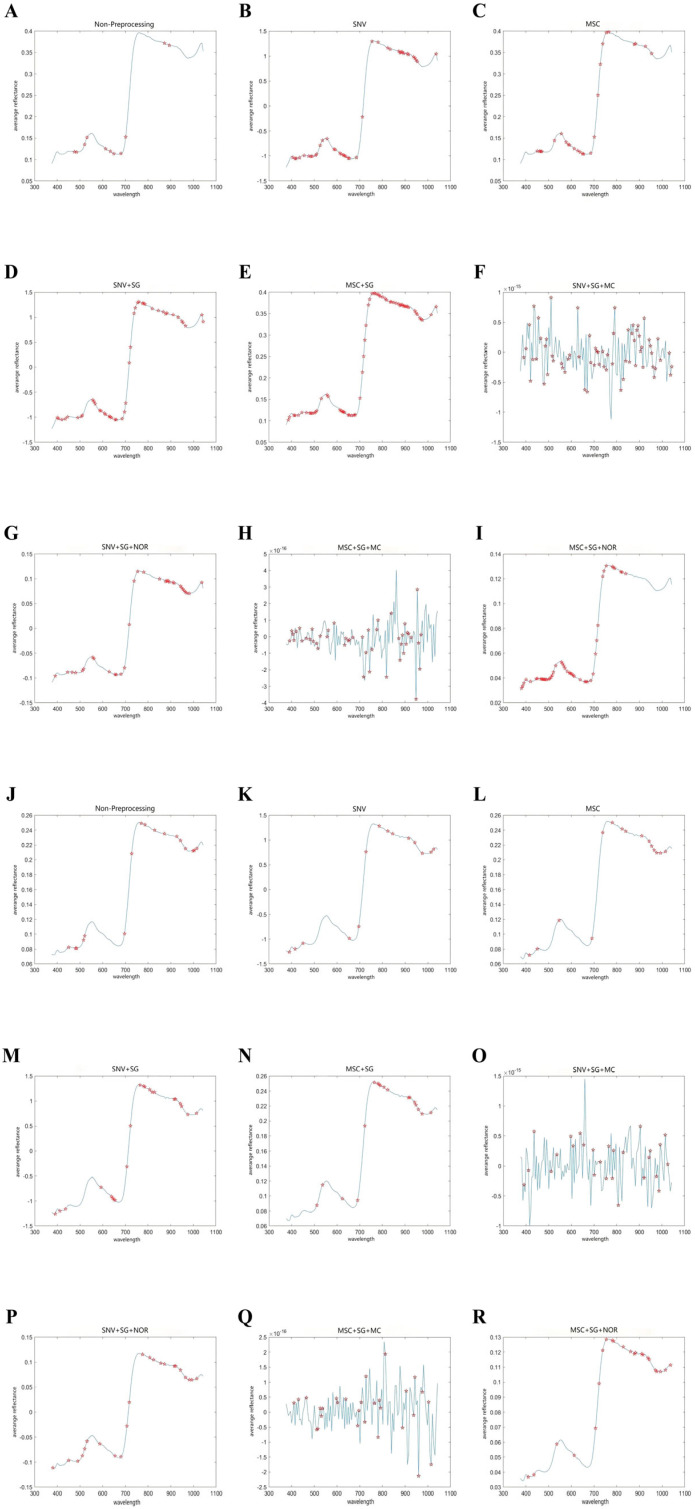
Extracting canopy reflectance characteristics band by CARS. ((**A**–**I**): the pretreatment of the spectral data following the potato inoculation with *R. solanacearum* at 0 h, 24 h, 48 h, and 72 h; (**J**–**R**): pretreatment of canopy spectral reflectance for different severity of potato bacterial wilt after inoculation with *R. solanacearum*).

The integration of CARS for feature selection was pivotal. It not only simplified the models but also improved their physiological interpretability. The selection of bands across the entire spectrum, particularly in the NIR after preprocessing (Figure 3), highlights the importance of both pigment-related (visible) and structural/water-related (NIR) information for early detection. This approach is more resource-efficient compared to full-spectrum analysis, echoing the principles of resource-efficient computing in modern agriculture.

Despite the high classification accuracies (exceeding 97%) achieved by the optimized PLS-DA models, it is important to interpret these results with caution due to the relatively limited sample size used in this study. The occurrence of perfect or near-perfect performance metrics, such as 100% sensitivity and precision on the test set, may indicate a risk of overfitting, particularly when the test set is small and the data are derived from a controlled experiment. Although Venetian blinds cross-validation was employed to mitigate overfitting during model training, this internal validation cannot fully guarantee generalizability to unseen data from different environments or genotypes.

### 3.2. Graded Detection of Potato Bacterial Wilt Severity

The progressive decrease in reflectance with increasing disease severity (Figure 1B) is consistent with known plant pathophysiology: chlorophyll degradation in the visible range (500–680 nm) and breakdown of leaf mesophyll structure in the NIR (750–1000 nm) [23,24]. The MSC + SG + MC + PLS-DA model effectively captured these spectral signatures, achieving high classification accuracy for severity grades. This objective grading system surpasses the subjectivity of manual scoring and is crucial for high-throughput germplasm screening in breeding programs [25]. The concentration of CARS-selected features in the NIR for severity grading (Figure 3) strongly implicates water content and cellular integrity as key indicators of disease progression.

### 3.3. Limitations and Future Perspectives

While the HSI-based models developed in this study demonstrated high accuracy in early detection and severity grading, it is important to acknowledge that disease assessment was primarily based on visual symptom scoring. Although visual scoring is a practical and widely used method for phenotyping, it remains inherently subjective and may not fully capture the underlying pathogen load or physiological changes at the tissue level. Reference methods such as quantitative PCR (qPCR) and bacterial colony counting (CFU) are considered gold standards for confirming infection and quantifying disease severity 7,12. The absence of such molecular and microbiological validation in the present study limits the direct biological interpretability of the spectral data. Future work should aim to integrate HSI with these laboratory-based assays to establish robust correlations between spectral signatures and actual pathogen burden, thereby enhancing the biological relevance and diagnostic reliability of the proposed approach. Despite these limitations, the demonstrated accuracy under controlled conditions provides a strong foundation for translating this approach to real-world agricultural settings.

Despite the promising results, several limitations should be acknowledged. First, the study relied on visual disease scoring, which is subjective and may not accurately reflect pathogen load. Reference methods such as qPCR and bacterial enumeration are needed to establish direct correlations between spectral signatures and actual disease intensity. Second, the use of a single susceptible genotype (‘Longshu No. 7’) under strictly controlled growth chamber conditions limits the generalizability of the models. Spectral signatures may vary among cultivars due to differences in leaf morphology, pigmentation, and canopy structure. Third, the monitoring window was limited to 72 h post-inoculation; extending the observation period would provide a more comprehensive view of spectral changes associated with advancing symptoms. Finally, field environments introduce additional sources of spectral noise, such as varying illumination, soil background, and co-occurring stresses. Therefore, model performance must be validated across a diverse panel of potato genotypes and in field environments. Addressing these limitations will be critical for translating HSI into practical agricultural applications.

The future trajectory of this research is aligned with the vision of Society 5.0 and smart agriculture. Integrating our HSI models with Unmanned Aerial Vehicles (UAVs) equipped with hyperspectral sensors presents a transformative opportunity for large-scale, real-time field monitoring [26,27]. This would enable the creation of disease hotspot maps for targeted intervention, optimizing resource use. Furthermore, embedding this technology within a cloud/fog computing architecture would facilitate real-time data analysis and decision-making, creating a comprehensive cyber-physical system for sustainable crop protection.

### 3.4. Comparison with Deep Learning Approaches

The present study employed traditional machine learning algorithms (PLS-DA and PCA-LDA) due to their interpretability, lower sample size requirements, and suitability for exploratory research. These methods have been widely used in hyperspectral plant disease detection and provide transparent models that are easier to validate and troubleshoot. However, deep learning, particularly convolutional neural networks (CNNs) and vision transformers, has recently demonstrated superior performance in extracting complex spatial–spectral features from hyperspectral images, often achieving higher accuracy in large-scale datasets [21,23,28]. The main trade-off lies in data demand and computational cost: deep learning models typically require thousands of labeled samples and powerful hardware, whereas traditional methods can yield robust results with smaller datasets. Given the controlled nature and moderate sample size of our experiment, PLS-DA and PCA-LDA were appropriate choices. Nevertheless, as HSI data accumulate and field deployments become more common, exploring hybrid CNN architectures or transfer learning may further enhance detection accuracy and automation. Future research should systematically compare these approaches under identical conditions to guide method selection for different application scenarios.

### 3.5. Field Application Potential and Future Plans

Although the hyperspectral imaging (HSI) techniques demonstrated high accuracy under controlled conditions, applying these methods in real-world field environments presents additional challenges. Factors such as varying sunlight intensity, soil reflectance, and atmospheric conditions can introduce noise and reduce the reliability of spectral measurements.

To address these challenges, future studies will explore integrating standardized light sources and adaptive calibration techniques to ensure data consistency. Moreover, the scalability of HSI for large-scale field applications is highly promising. By mounting hyperspectral sensors on unmanned aerial vehicles (UAVs) or autonomous ground-based platforms, this technology could facilitate rapid, high-throughput disease monitoring across extensive agricultural landscapes. Such systems would enable the identification of disease hotspots, allowing for targeted interventions and efficient resource allocation.

To validate the robustness of the models, a series of field trials will be conducted across diverse geographic regions and climatic conditions. These trials will focus on evaluating the detection and grading accuracy of HSI for various potato genotypes and under natural pathogen pressure. Additionally, the potential to integrate HSI with other precision agriculture tools, such as soil moisture sensors and weather data models, will be explored to enhance the predictive power of disease management systems.

From a computational perspective, the term “rapid detection” used throughout this study refers primarily to the speed of data acquisition—canopy reflectance can be captured within seconds using a portable hyperspectral imager. However, the full processing pipeline, including data preprocessing, feature extraction (e.g., CARS), and model classification, currently requires offline computation and is not yet optimized for real-time field deployment. With the rapid advancement of edge computing devices and optimized algorithms, it is increasingly feasible to accelerate these processing steps to achieve near-real-time or on-the-go analysis. For instance, implementing lightweight versions of CARS and PLS-DA on embedded systems, or deploying compact deep learning models on UAV-mounted hardware, could enable instantaneous disease mapping during field inspections.

Another important consideration is the cost-effectiveness of HSI technology. Hyperspectral imagers remain relatively expensive compared to multispectral or RGB cameras, which may limit their widespread adoption in routine farming practices. Nevertheless, for high-value crops such as potato, and for applications in breeding programs and precision agriculture research, the rich spectral information provided by HSI justifies the investment by enabling early disease intervention and reducing unnecessary pesticide applications. As sensor technology continues to advance and production costs decline, HSI is expected to become more accessible to growers and agricultural extension services. Future research should include a comprehensive cost–benefit analysis that accounts for potential savings from reduced yield losses, optimized chemical inputs, and improved resistance screening efficiency. Such analysis would strengthen the economic case for adopting HSI-based disease monitoring as a routine tool in integrated pest management strategies. Beyond the practical considerations of field deployment and cost-effectiveness, the choice of analytical methodology also plays a critical role in determining the accuracy and scalability of HSI-based diagnostics. In this context, a comparison between traditional machine learning and emerging deep learning approaches warrants further discussion.

In addition to expanding the field applicability of HSI, future studies should also focus on extending the temporal monitoring window beyond the early infection stages examined here (up to 72 h post-inoculation). Monitoring disease progression over a longer period—such as 7 to 14 days post-inoculation—would provide a more comprehensive view of spectral changes associated with advancing symptoms and tissue degradation. This would not only improve the robustness of severity grading models but also enable the identification of spectral biomarkers associated with late-stage infection.

Furthermore, to strengthen the biological validity of HSI-based diagnostics, it is essential to integrate conventional laboratory methods such as qPCR and bacterial enumeration (CFU counts) into the experimental design. These quantitative assays can provide an objective measure of pathogen load and disease intensity, allowing for direct calibration and validation of spectral data. Such a multi-modal approach—combining hyperspectral imaging with molecular and microbiological tools—would significantly enhance the accuracy, interpretability, and translational value of disease detection models in both controlled and field environments.

## 4. Materials and Methods

### 4.1. Plant Material and Experimental Design

The study used ‘Longshu No. 7’, a winter potato variety in South China highly susceptible to bacterial wilt. A total of 160 potato plants were grown individually in pots (15 cm × 15 cm) filled with a vermiculite:peat soil (3:1) substrate in an artificial climate chamber. The chamber conditions were maintained at 23 °C/22 °C (day/night) with a 16-h light/8-h dark cycle. Before inoculation, plants were acclimatized at 32/30 °C for 5 days [29]. This setup provided 160 biological replicates. Each plant was considered an independent experimental unit.

### 4.2. Experimental Workflow

The experimental workflow comprised the following main stages: (1) cultivation of potato plants (‘Longshu No. 7’) under controlled conditions; (2) inoculation with *R. solanacearum* (strain P2) using the root injury method; (3) hyperspectral image acquisition at 0, 24, 48, and 72 h post-inoculation (hpi); (4) visual assessment of disease severity using a 0–4 scale; (5) extraction of canopy reflectance spectra and preprocessing using various techniques (SNV, MSC, SG, MC, NOR); (6) feature band selection using Competitive Adaptive Reweighted Sampling (CARS); (7) development of classification models (PLS-DA and PCA-LDA) for early detection and severity grading; and (8) model evaluation using independent test sets and cross-validation.

### 4.3. Plant Inoculation

The *R. solanacearum* P2 strain (phylotype I), isolated from infected potatoes in Guangdong, China, was used for inoculation. Bacteria were cultured overnight, centrifuged, and adjusted to a concentration of 10^7^ CFU/mL. Potato plants were inoculated using the root injury method [30] and subsequently placed in a controlled environment chamber at 32/30 °C.

**Figure 4:**
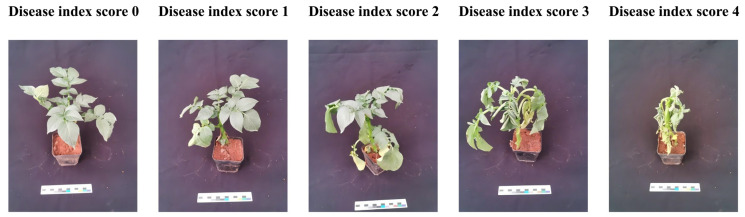
The disease severity of the potato bacterial wilt after inoculation with *R. solanacearum*.

Disease severity was assessed visually at each time point using a 0–4 scale based on the percentage of wilted leaves, as illustrated in Figure 4: grade 0 = no wilting symptoms; grade 1 = 1–25% of leaves wilted; grade 2 = 26–50%; grade 3 = 51–75%; grade 4 = 76–100% wilting or plant death. This scale is commonly used in potato bacterial wilt phenotyping [31] and provides a practical measure of disease progression. However, visual scoring remains inherently subjective and may not accurately reflect the internal pathogen load. Future studies should incorporate quantitative assays, such as bacterial colony counting (CFU/g tissue) or quantitative PCR (qPCR) targeting *R. solanacearum*-specific genes, to establish a more objective ground truth for severity grading and to validate the spectral patterns observed (Figure 4) [31,32]. Non-inoculated control plants were maintained under identical growth chamber conditions throughout the experiment. These plants were subjected to the same handling procedures as inoculated plants, including mock inoculation using sterile water, and were measured at the same time points (0, 24, 48, and 72 hpi). Spectral data from these controls were used to confirm that observed changes in inoculated plants were attributable to *R. solanacearum* infection rather than environmental fluctuations or handling stress.

### 4.4. Hyperspectral Image Acquisition and Data Collection Strategy

**Figure 5:**
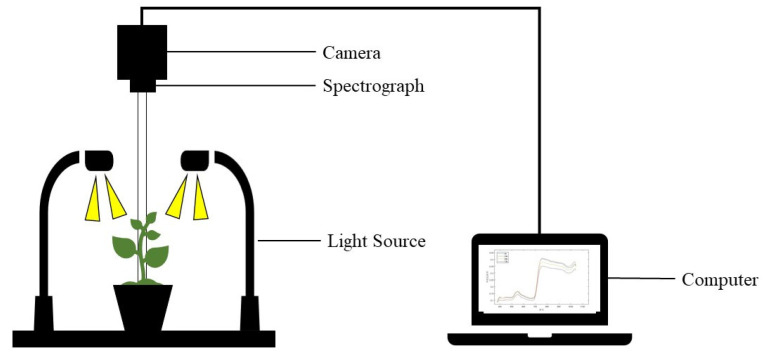
Schematic of the hyperspectral imaging system for acquiring spectral scattering images from potato plants.

Hyperspectral data were collected in a darkroom using a SOC710VP portable hyperspectral imager (400–1000 nm spectral range, 1.3 nm resolution, 128 spectral bands). The imaging setup (Figure 5) was standardized: the camera was positioned vertically 50 cm above the plant canopy, illuminated by two 500-W halogen lamps at a 45° angle to minimize specular reflection. Exposure time was set to 15 ms. Reflectance calibration was performed using a built-in white reference panel before each session. Spectral data were collected from each of the 160 plants at 0, 24, 48, and 72 h post-inoculation (hpi), resulting in 640 spectral observations. For model development, data from each time point (*n* = 160 observations per time point) were randomly split into a training set (*n* = 128, 80%) and a testing set (*n* = 32, 20%). Similarly, for severity grading, all plants at the time points when symptoms appeared were pooled and split into training and testing sets based on their assigned disease grade.

### 4.5. Processing of Hyperspectral Data

The hyperspectral data processing involved three main steps: (1) reflectance conversion and region of interest (ROI) extraction, (2) spectral preprocessing, and (3) feature extraction and classification modeling.

#### 4.5.1. Reflectance Conversion and ROI Extraction

Raw digital number (DN) values were converted to reflectance using SRAnal(710) software. The average reflectance from the entire plant canopy was extracted as the Region of Interest (ROI) using ENVI 5.3.

#### 4.5.2. Preprocessing of Hyperspectral Data

Reflectance data were preprocessed in MATLAB R2020a using eight different methods, individually or in combination (Table 5). Key parameters are specified: Savitzky–Golay (SG) smoothing was applied with a second-order polynomial and a window size of 11 points; Normalization (NOR) was performed relative to the band at 800 nm.

#### 4.5.3. Feature Variables Extraction

Competitive Adaptive Reweighted Sampling (CARS) was employed to select informative wavelengths, reducing data dimensionality. The CARS parameters were set as follows: Monte Carlo sampling runs: 50; Threshold (α): 0.05.

#### 4.5.4. Classification and Discrimination

Two distinct analytical workflows were implemented: one for early detection (classifying samples into 0 h, 24 h, 48 h, 72 h) and another for severity grading (classifying samples into disease levels 0–4). For each, PLS-DA and PCA-LDA models were constructed using the Classification Toolbox 6.0. The number of latent variables (LVs) for PLS-DA was optimized via cross-validation. PCA-LDA retained principal components explaining >99% variance. Model evaluation employed Venetian Blinds cross-validation (10 data splits) on the training set. Performance was assessed on the independent test set using Accuracy, Precision, Sensitivity, and Specificity. Confusion matrices were generated for all final models to provide detailed insight into classification performance.

## 5. Conclusions

This study demonstrates that hyperspectral imaging, combined with optimized PLS-DA models and CARS feature selection, enables two critical advances: (1) early detection of potato bacterial wilt as early as 24 h post-inoculation, before visible symptoms appear; and (2) objective, high-accuracy grading of disease severity (grades 0–4). The ability to detect infection pre-symptomatically and quantify disease progression objectively represents a paradigm shift from reactive to proactive disease management. This work provides a robust framework for high-throughput resistance screening in breeding programs and lays the groundwork for integrating HSI with UAV and IoT-based platforms for precision agriculture, ultimately contributing to enhanced potato productivity and global food security.

## Figures and Tables

**Table 1:** The pretreatment of the spectral.

	Preprocessing Method
1	Non-Preprocessing
2	SNV (Standard Normalized Variate)
3	MSC (Multiple scattering correction)
4	SNV (Standard Normalized Variate) + SG (Savitzky-Golay Convolutional)
5	MSC (Multiple scattering correction) + SG (Savitzky-Golay Convolutional)
6	SNV (Standard Normalized Variate) + SG (Savitzky-Golay Convolutional) + MC (Mean Centering)
7	SNV (Standard Normalized Variate) + SG (Savitzky-Golay Convolutional) + NOR (Normalize)
8	MSC (Multiple scattering correction) + SG (Savitzky-Golay Convolutional) + MC (Mean Centering)
9	MSC (Multiple scattering correction) + SG (Savitzky-Golay Convolutional) + NOR (Normalize)

**Table 2:** Early detection and classification of potato bacterial wilt severity using canopy spectral reflectance and PLS-DA model.

Pre-Processing Technique	The Early Detection of Potato Bacterial Wilt Inoculation									The Classification of Potato Bacterial Wilt Disease Severity								
	Hours Post Inoculation	CV Detection of Training Data Set (%)				Test Data Set (%)				Disease Score	CV Detection of Training Data Set (%)				Test Data Set (%)			
		Accuracy	Sensitivity	Specificity	Precision	Accuracy	Sensitivity	Specificity	Precision		Accuracy	Sensitivity	Specificity	Precision	Accuracy	Sensitivity	Specificity	Precision
1	0 h	87.00	100.00	96.00	84.00	87.00	100.00	95.00	90.00	0	72.00	94.00	100.00	100.00	86.00	100.00	100.00	100.00
	24 h		83.00	97.00	91.00		86.00	96.00	86.00	1		55.00	92.00	69.00		100.00	100.00	100.00
	48 h		79.00	94.00	85.00		67.00	100.00	100.00	2		60.00	92.00	60.00		50.00	89.00	33.00
	72 h		90.00	94.00	87.00		89.00	91.00	80.00	3		76.00	88.00	62.00		67.00	94.00	67.00
										4		78.00	93.00	74.00		67.00	100.00	100.00
2	0 h	79.00	92.00	95.00	86.00	79.00	100.00	100.00	100.00	0	71.00	90.00	93.00	83.00	63.00	75.00	100.00	100.00
	24 h		76.00	91.00	76.00		75.00	92.00	75.00	1		46.00	94.00	60.00		100.00	85.00	60.00
	48 h		69.00	91.00	74.00		63.00	88.00	63.00	2		33.00	93.00	38.00		50.00	86.00	33.00
	72 h		81.00	94.00	81.00		78.00	92.00	78.00	3		71.00	90.00	67.00		40.00	91.00	67.00
										4		88.00	95.00	82.00		50.00	93.00	50.00
3	0 h	84.00	96.00	96.00	89.00	91.00	100.00	100.00	100.00	0	70.00	90.00	93.00	83.00	70.00	75.00	100.00	100.00
	24 h		81.00	95.00	86.00		89.00	100.00	100.00	1		46.00	90.00	50.00		100.00	83.00	60.00
	48 h		74.00	94.00	80.00		75.00	96.00	86.00	2		25.00	94.00	33.00		50.00	85.00	33.00
	72 h		85.00	93.00	79.00		100.00	92.00	78.00	3		69.00	90.00	65.00		25.00	91.00	50.00
										4		83.00	95.00	83.00		50.00	92.00	50.00
4	0 h	99.00	100.00	100.00	100.00	88.00	100.00	100.00	100.00	0	88.00	100.00	99.00	97.00	83.00	100.00	100.00	100.00
	24 h		100.00	100.00	100.00		80.00	95.00	89.00	1		79.00	95.00	75.00		100.00	95.00	89.00
	48 h		97.00	100.00	100.00		83.00	92.00	71.00	2		73.00	97.00	79.00		25.00	96.00	50.00
	72 h		100.00	99.00	97.00		88.00	96.00	88.00	3		81.00	97.00	85.00		75.00	95.00	86.00
										4		96.00	99.00	96.00		100.00	93.00	50.00
5	0 h	99.00	100.00	100.00	100.00	90.00	100.00	100.00	100.00	0	84.00	90.00	99.00	97.00	76.00	86.00	100.00	100.00
	24 h		100.00	100.00	100.00		88.00	95.00	88.00	1		79.00	94.00	71.00		100.00	100.00	100.00
	48 h		97.00	100.00	100.00		83.00	96.00	83.00	2		78.00	95.00	74.00		33.00	89.00	33.00
	72 h		100.00	99.00	97.00		86.00	95.00	86.00	3		79.00	93.00	79.00		80.00	94.00	80.00
										4		90.00	99.00	95.00		50.00	89.00	33.00
6	0 h	98.00	100.00	98.00	94.00	97.00	100.00	100.00	100.00	0	88.00	100.00	99.00	97.00	82.00	100.00	100.00	100.00
	24 h		94.00	100.00	100.00		100.00	100.00	100.00	1		77.00	97.00	87.00		100.00	100.00	100.00
	48 h		100.00	100.00	100.00		88.00	100.00	100.00	2		81.00	96.00	84.00		75.00	83.00	43.00
	72 h		100.00	100.00	100.00		100.00	96.00	89.00	3		86.00	94.00	77.00		50.00	100.00	100.00
										4		92.00	98.00	92.00		100.00	96.00	75.00
7	0 h	99.00	100.00	100.00	100.00	91.00	100.00	100.00	100.00	0	82.00	89.00	97.00	89.00	83.00	86.00	100.00	100.00
	24 h		100.00	100.00	100.00		80.00	95.00	89.00	1		75.00	94.00	71.00		100.00	100.00	100.00
	48 h		97.00	100.00	100.00		83.00	92.00	71.00	2		63.00	97.00	83.00		67.00	93.00	67.00
	72 h		100.00	99.00	97.00		88.00	96.00	88.00	3		86.00	90.00	74.00		100.00	87.00	60.00
										4		95.00	99.00	95.00		0.00	100.00	0.00
8	0 h	98.00	100.00	98.00	94.00	94.00	100.00	100.00	100.00	0	92.00	100.00	99.00	97.00	82.00	100.00	100.00	100.00
	24 h		94.00	100.00	100.00		100.00	95.00	91.00	1		82.00	99.00	95.00		80.00	100.00	100.00
	48 h		100.00	100.00	100.00		86.00	100.00	100.00	2		90.00	97.00	86.00		67.00	88.00	40.00
	72 h		100.00	100.00	100.00		88.00	96.00	88.00	3		92.00	95.00	81.00		57.00	95.00	80.00
										4		92.00	100.00	100.00		100.00	96.00	80.00
9	0 h	99.00	100.00	100.00	100.00	88.00	100.00	100.00	100.00	0	88.00	100.00	99.00	97.00	85.00	100.00	100.00	100.00
	24 h		100.00	100.00	100.00		80.00	95.00	89.00	1		71.00	99.00	94.00		100.00	95.00	83.00
	48 h		97.00	100.00	100.00		83.00	92.00	71.00	2		81.00	96.00	76.00		100.00	88.00	40.00
	72 h		100.00	99.00	97.00		88.00	96.00	88.00	3		86.00	92.00	77.00		63.00	100.00	100.00
										4		95.00	99.00	95.00		75.00	100.00	100.00

**Table 3:** Early detection and classification of potato bacterial wilt severity using canopy spectral reflectance and PCA-LDA model.

	The Early Detection of Potato Bacterial Wilt Inoculation									The Classification of Potato Bacterial Wilt Disease Severity								
Pre-Processing Technique	Hours Post Inoculation	CV Detection of Training Data Set (%)				Test Data Set (%)				Disease Score	CV Detection of Training Data Set (%)				Test Data Set (%)			
		Accuracy	Sensitivity	Specificity	Precision	Accuracy	Sensitivity	Specificity	Precision		Accuracy	Sensitivity	Specificity	Precision	Accuracy	Sensitivity	Specificity	Precision
1	0 h	80.00	91.00	97.00	91.00	75.00	100.00	97.00	91.00	0	71.00	81.00	97.00	85.00	70.00	70.00	97.00	88.00
	24 h		85.00	93.00	80.00		60.00	93.00	75.00	1		57.00	93.00	68.00		90.00	90.00	69.00
	48 h		65.00	91.00	72.00		60.00	87.00	60.00	2		67.00	89.00	62.00		70.00	85.00	54.00
	72 h		81.00	92.00	76.00		80.00	90.00	73.00	3		74.00	89.00	62.00		50.00	95.00	71.00
										4		77.00	95.00	81.00		70.00	95.00	78.00
2	0 h	84.00	94.00	96.00	86.00	72.00	100.00	97.00	91.00	0	70.00	75.00	95.00	77.00	64.00	50.00	97.00	83.00
	24 h		85.00	95.00	87.00		50.00	93.00	71.00	1		62.00	91.00	64.00		80.00	82.00	53.00
	48 h		72.00	95.00	85.00		60.00	80.00	50.00	2		64.00	90.00	63.00		70.00	85.00	54.00
	72 h		89.00	93.00	80.00		80.00	93.00	80.00	3		71.00	89.00	63.00		50.00	95.00	71.00
										4		79.00	97.00	89.00		70.00	95.00	78.00
3	0 h	82.00	94.00	97.00	91.00	25.00	0.00	100.00	0.00	0	69.00	81.00	94.00	76.00	20.00	0.00	100.00	0.00
	24 h		85.00	91.00	77.00		100.00	0.00	25.00	1		59.00	92.00	65.00		100.00	0.00	20.00
	48 h		68.00	93.00	77.00		0.00	100.00	0.00	2		62.00	90.00	62.00		0.00	100.00	0.00
	72 h		83.00	95.00	83.00		0.00	100.00	0.00	3		66.00	87.00	57.00		0.00	100.00	0.00
										4		77.00	97.00	88.00		0.00	100.00	0.00
4	0 h	93.00	97.00	97.00	91.00	85.00	80.00	100.00	100.00	0	85.00	86.00	97.00	89.00	78.00	90.00	100.00	100.00
	24 h		92.00	98.00	95.00		100.00	93.00	83.00	1		78.00	95.00	78.00		100.00	95.00	83.00
	48 h		90.00	97.00	92.00		90.00	90.00	75.00	2		90.00	94.00	80.00		80.00	82.00	53.00
	72 h		92.00	97.00	92.00		70.00	97.00	88.00	3		79.00	95.00	79.00		60.00	100.00	100.00
										4		90.00	100.00	100.00		60.00	95.00	75.00
5	0 h	94.00	97.00	98.00	94.00	25.00	0.00	100.00	0.00	0	84.00	83.00	97.00	86.00	20.00	0.00	100.00	0.00
	24 h		95.00	98.00	95.00		0.00	100.00	0.00	1		65.00	95.00	77.00		0.00	100.00	0.00
	48 h		93.00	97.00	93.00		100.00	0.00	25.00	2		95.00	94.00	80.00		0.00	100.00	0.00
	72 h		92.00	98.00	94.00		0.00	100.00	0.00	3		87.00	93.00	77.00		0.00	100.00	0.00
										4		87.00	100.00	100.00		100.00	0.00	20.00
6	0 h	95.00	100.00	98.00	94.00	82.00	90.00	100.00	100.00	0		86.00	97.00	89.00		90.00	100.00	100.00
	24 h		95.00	100.00	100.00		90.00	90.00	75.00	1		78.00	95.00	78.00		100.00	93.00	77.00
	48 h		93.00	97.00	93.00		60.00	93.00	75.00	2	85.00	90.00	94.00	80.00	76.00	80.00	82.00	53.00
	72 h		92.00	97.00	92.00		90.00	93.00	82.00	3		79.00	95.00	79.00		50.00	100.00	100.00
										4		90.00	100.00	100.00		60.00	95.00	75.00
7	0 h	94.00	97.00	98.00	94.00	85.00	90.00	100.00	100.00	0	84.00	89.00	97.00	86.00	76.00	90.00	100.00	100.00
	24 h		95.00	98.00	95.00		80.00	97.00	89.00	1		78.00	95.00	78.00		90.00	95.00	82.00
	48 h		90.00	98.00	95.00		70.00	87.00	64.00	2		87.00	94.00	79.00		80.00	82.00	53.00
	72 h		94.00	97.00	92.00		80.00	90.00	73.00	3		79.00	95.00	79.00		60.00	97.00	86.00
										4		87.00	100.00	100.00		60.00	95.00	75.00
8	0 h	95.00	100.00	98.00	94.00	85.00	90.00	100.00	100.00	0	85.00	90.00	100.00	100.00	82.00	90.00	100.00	100.00
	24 h		95.00	100.00	100.00		100.00	90.00	77.00	1		100.00	97.00	91.00		100.00	97.00	91.00
	48 h		93.00	97.00	93.00		60.00	97.00	86.00	2		90.00	85.00	60.00		90.00	85.00	60.00
	72 h		92.00	97.00	92.00		90.00	93.00	82.00	3		70.00	97.00	88.00		70.00	97.00	88.00
										4		60.00	97.00	86.00		60.00	97.00	86.00
9	0 h	94.00	97.00	98.00	94.00	82.00	80.00	100.00	100.00	0	84.00	89.00	97.00	89.00	78.00	80.00	100.00	100.00
	24 h		95.00	98.00	95.00		100.00	93.00	83.00	1		78.00	95.00	78.00		100.00	93.00	77.00
	48 h		90.00	98.00	95.00		70.00	93.00	78.00	2		87.00	94.00	79.00		80.00	85.00	57.00
	72 h		94.00	97.00	92.00		80.00	90.00	73.00	3		79.00	94.00	77.00		60.00	100.00	100.00
										4		87.00	100.00	100.00		70.00	95.00	78.00

**Table 4:** Extraction of Canopy Reflectance Characteristic Bands at Early Stages and Disease Severity Levels of Potatoes Inoculated with *Ralstonia solanacearum* Using CARS.

	The Early Detection of Potato Bacterial Wilt Inoculation			The Classification of Potato Bacterial Wilt Disease Severity		
Pre-Processing Technique	Specific Wavelengths	minRMSECV	Optimal Sampling Times	Specific Wavelengths	minRMSECV	Optimal Sampling Times
1	475.34, 485.40, 520.77, 530.92, 612.87, 633.56, 649.14, 680.42, 701.38, 872.02, 893.72	0.46	22	480.37, 520.77, 711.89, 722.42, 738.26, 786.04, 866.61, 926.42, 975.82, 1003.44, 1014.53, 1020.08	0.5	25
2	405.50, 415.42, 420.38, 435.31, 455.28, 475.34, 485.40, 490.44, 505.58, 510.64, 525.84, 536.00, 556.39, 587.12, 592.26, 612.87, 618.04, 628.38, 633.56, 638.75, 649.14, 654.34, 685.66, 711.89, 754.14, 780.71, 823.50, 834.24, 872.02, 877.44, 882.86, 888.29, 893.72, 910.05, 915.50, 937.36, 942.84, 948.33, 953.81, 1036.77	0.4	4	390.67, 415.42, 450.28, 654.34, 696.14, 727.70, 786.04, 823.50, 845.01, 915.05, 942.84, 1014.53, 1025.64	0.49	26
3	390.67, 405.50, 420.38, 465.30, 480.37, 485.40, 490.44, 505.58, 510.64, 525.84, 546.19, 551.28, 561.50, 587.12, 592.26, 623.21, 628.38, 633.56, 643.94, 649.14, 654.34, 685.66, 701.38, 711.89, 738.26, 754.14, 764.76, 818.13, 834.24, 850.40, 877.44, 882.86, 893.72, 904.60, 910.05, 915.50, 926.42, 964.81, 970.31, 975.82, 997.91	0.37	4	410.46, 440.30, 536.00, 696.14, 743.55, 780.71, 786.04, 791.38, 807.41, 818.13, 828.87, 915.50, 942.84, 975.82, 981.34	0.54	9
4	400.55, 405.50, 420.38, 435.31, 445.29, 490.44, 505.58, 510.64, 556.39, 561.50, 566.61, 587.12, 592.26, 607.71, 612.87, 628.38, 633.56, 638.75, 654.34, 659.54, 685.66, 696.14, 701.38, 717.16, 722.42, 738.26, 743.55, 754.14, 759.45, 775.39, 780.71, 786.04, 818.13, 845.01, 866.61, 877.44, 882.86, 910.05, 931.89, 948.33, 953.81, 964.81, 1036.77, 1042.34	0.25	4	390.67, 410.46, 435.31, 592.26, 638.75, 643.94, 649.14, 654.34, 706.64, 722.42, 764.76, 780.71, 786.04, 807.41, 818.13, 828.87, 915.50, 920.96, 942.84, 948.33, 975.82, 1014.53	0.33	10
5	385.73, 390.67, 410.46, 415.42, 430.33, 445.29, 465.30, 480.37, 485.40, 490.44, 500.53, 505.58, 510.64, 530.92, 556.39, 561.50, 612.87, 618.04, 623.21, 628.38, 633.56, 649.14, 654.34, 664.76, 675.20, 680.42, 701.38, 711.89, 717.16, 722.42, 727.70, 738.26, 743.55, 754.14, 764.76, 770.07, 780.71, 786.04, 791.38, 802.06, 812.77, 818.13, 834.24, 839.62, 850.40, 861.20, 872.02, 877.44, 882.86, 888.29, 893.72, 899.16, 904.60, 910.05, 915.50, 942.84, 948.33, 953.81, 964.81, 970.31, 975.82, 1014.53, 1036.77	0.23	2	510.64, 536.00, 623.21, 690.89, 722.42, 764.76, 780.71, 786.04, 791.38, 807.41, 823.50, 915.50, 920.96, 942.84, 948.33, 959.31, 975.82, 1014.53	0.34	17
6	380.80, 385.73, 415.42, 420.38, 430.33, 435.31, 450.28, 455.28, 465.30, 480.37, 485.40, 490.44, 495.48, 505.58, 510.64, 525.84, 546.19, 556.39, 561.50, 587.12, 597.40, 602.55, 607.71, 612.87, 633.56, 638.75, 680.42, 685.66, 717.16, 722.42, 738.26, 743.55, 754.14, 759.45, 770.07, 780.71, 786.04, 818.13, 834.24, 839.62, 850.40, 882.86, 888.29, 893.72, 910.05, 915.50, 920.96, 926.42, 931.89, 948.33, 953.81, 964.81, 1008.99, 1036.77	0.28	3	390.67, 410.46, 435.31, 510.64, 536.00, 597.40, 607.71, 638.75, 654.34, 696.14, 701.38, 727.70, 754.14, 764.76, 780.71, 786.04, 807.41, 827.87, 904.5989, 920.958, 942.842, 948.3258, 975.822, 986.8564, 992.3813, 1014.5323, 1025.6385	0.34	8
7	500.53, 505.58, 520.77, 546.19, 556.39, 587.12, 628.38, 643.94, 649.14, 659.54, 669.97, 711.89, 738.26, 759.45, 770.07, 786.04, 796.72, 812.77, 823.50, 839.62, 845.01, 888.29, 893.72, 948.33, 953.81	0.26	9	380.8043, 450.2831, 490.437, 510.6372, 520.7681, 530.9195, 587.1194, 654.3383, 680.4228, 706.6356, 717.1566, 775.3893, 807.4145, 823.4964, 855.7988, 872.0193, 915.4998, 920.958, 942.842, 964.8081, 981.3367, 992.3813, 1014.5323	0.37	12
8	420.38, 430.33, 435.31, 465.30, 475.34, 480.37, 490.44, 505.58, 510.64, 515.70, 525.84, 530.92, 556.39, 561.50, 566.61, 571.73, 587.12, 607.71, 628.38, 633.56, 638.75, 649.14, 680.42, 685.66, 711.89, 738.26, 754.14, 759.45, 775.39, 780.71, 791.38, 818.13, 855.80, 866.61, 872.02, 877.44, 882.86, 888.29, 893.72, 899.16, 910.05, 915.50, 926.42, 931.89, 937.36, 942.84, 948.33, 953.81, 964.81, 992.38, 997.91, 1036.77	0.27	3	410.46, 430.33, 465.30, 510.64, 515.70, 525.84, 530.92, 536.00, 597.40, 602.55, 638.75, 690.89, 696.14, 706.64, 722.42, 727.70, 764.76, 780.71, 786.04, 791.38, 812.77, 888.29, 904.60, 937.36, 942.84, 959.31, 975.82, 1003.45, 1014.53	0.36	7
9	380.80, 385.73, 390.67, 400.55, 420.38, 445.29, 450.28, 465.30, 470.32, 475.34, 480.37, 485.40, 490.44, 500.53, 505.58, 510.64, 515.70, 520.77, 530.92, 556.39, 561.50, 566.61, 571.73, 581.98, 592.26, 597.40, 602.55, 607.71, 612.87, 638.75, 659.54, 664.76, 669.97, 685.66, 696.14, 706.64, 711.89, 717.16, 738.26, 743.55, 754.14, 780.71, 786.04, 791.38, 818.13, 823.50, 839.62, 850.40, 872.02, 877.44, 888.29, 893.72, 904.60, 910.05, 915.50, 937.36, 942.84, 948.33, 953.81, 964.81, 981.34, 992.38, 1008.99, 1014.53, 1042.34	0.27	2	410.46, 435.31, 536.00, 612.87, 706.64, 722.42, 738.26, 754.14, 780.71, 786.04, 828.87, 861.20, 877.44, 882.86, 910.05, 915.50, 937.36, 942.84, 970.31, 975.82, 992.38, 1014.53, 1036.77	0.36	9

**Table 5:** Early Infection Detection and Severity Grading of Potato Bacterial Wilt Using Characteristic Bands Extracted by CARS and the PLS-DA Model.

Pre-Processing Technique	Hours Post Inoculation	CV Detection of Training Data Set (%)				Test Data Set (%)				Disease Score	CV Detection of Training Data Set (%)				Test Data Set (%)			
		Accuracy	Sensitivity	Specificity	Precision	Accuracy	Sensitivity	Specificity	Precision		Accuracy	Sensitivity	Specificity	Precision	Accuracy	Sensitivity	Specificity	Precision
1	0 h	77.00	94.00	97.00	88.00	75.00	100.00	100.00	100.00	0	60.00	78.00	94.00	70.00	69.00	50.00	82.00	33.00
	24 h		81.00	89.00	78.00		60.00	82.00	60.00	1		45.00	90.00	50.00		60.00	88.00	75.00
	48 h		58.00	90.00	65.00		60.00	82.00	60.00	2		38.00	90.00	50.00		50.00	100.00	100.00
	72 h		78.00	93.00	78.00		100.00	100.00	100.00	3		63.00	85.00	59.00		100.00	100.00	100.00
										4		79.00	90.00	69.00		100.00	91.00	67.00
2	0 h	78.00	96.00	95.00	83.00	83.00	100.00	100.00	100.00	0	70.00	89.00	96.00	89.00	79.00	100.00	100.00	100.00
	24 h		73.00	90.00	71.00		60.00	92.00	60.00	1		57.00	88.00	53.00		50.00	100.00	100.00
	48 h		61.00	96.00	88.00		78.00	86.00	70.00	2		40.00	93.00	50.00		100.00	85.00	33.00
	72 h		88.00	89.00	70.00		88.00	100.00	100.00	3		75.00	93.00	75.00		67.00	88.00	80.00
										4		75.00	93.00	69.00		100.00	100.00	100.00
3	0 h	79.00	95.00	95.00	83.00	57.00	100.00	100.00	100.00	0	65.00	93.00	97.00	87.00	71.00	100.00	93.00	67.00
	24 h		75.00	92.00	80.00		0.00	100.00	0.00	1		62.00	92.00	62.00		100.00	73.00	67.00
	48 h		64.00	93.00	81.00		100.00	14.00	54.00	2		29.00	86.00	33.00		NaN	94.00	0.00
	72 h		91.00	91.00	71.00		0.00	100.00	0.00	3		75.00	85.00	65.00		33.00	100.00	100.00
										4		67.00	97.00	80.00		67.00	100.00	100.00
4	0 h	97.00	100.00	99.00	96.00	100.00	100.00	100.00	100.00	0	79.00	86.00	98.00	86.00	91.00	0.00	100.00	0.00
	24 h		94.00	100.00	100.00		100.00	100.00	100.00	1		67.00	92.00	33.00		100.00	100.00	100.00
	48 h		94.00	99.00	97.00		100.00	100.00	100.00	2		76.00	90.00	76.00		80.00	100.00	100.00
	72 h		100.00	98.00	94.00		100.00	100.00	100.00	3		75.00	98.00	90.00		100.00	89.00	67.00
										4		82.00	95.00	88.00		100.00	100.00	100.00
5	0 h	97.00	97.00	99.00	97.00	36.00	0.00	100.00	0.00	0	79.00	100.00	96.00	86.00	85.00	100.00	100.00	100.00
	24 h		97.00	99.00	97.00		100.00	0.00	36.00	1		68.00	97.00	88.00		0.00	100.00	0.00
	48 h		97.00	99.00	97.00		0.00	100.00	0.00	2		56.00	94.00	67.00		0.00	100.00	0.00
	72 h		97.00	99.00	97.00		0.00	100.00	0.00	3		75.00	91.00	63.00		100.00	100.00	100.00
										4		95.00	96.00	86.00		100.00	82.00	50.00
6	0 h	98.00	100.00	99.00	97.00	86.00	100.00	100.00	100.00	0	83.00	100.00	100.00	100.00	76.00	100.00	100.00	100.00
	24 h		97.00	100.00	100.00		83.00	91.00	71.00	1		83.00	96.00	83.00		60.00	100.00	100.00
	48 h		100.00	99.00	97.00		78.00	95.00	88.00	2		71.00	94.00	74.00		75.00	90.00	60.00
	72 h		97.00	100.00	100.00		83.00	95.00	83.00	3		74.00	91.00	69.00		71.00	83.00	63.00
										4		88.00	98.00	92.00		50.00	96.00	50.00
7	0 h	98.00	100.00	100.00	100.00	86.00	100.00	100.00	100.00	0	87.00	100.00	99.00	97.00	89.00	100.00	100.00	100.00
	24 h		100.00	100.00	100.00		50.00	100.00	100.00	1		79.00	97.00	83.00		100.00	93.00	80.00
	48 h		97.00	99.00	97.00		100.00	82.00	63.00	2		69.00	97.00	79.00		0.00	100.00	0.00
	72 h		97.00	99.00	97.00		86.00	100.00	100.00	3		81.00	93.00	74.00		83.00	100.00	100.00
										4		94.00	99.00	94.00		100.00	94.00	50.00
8	0 h	98.00	100.00	99.00	97.00	97.00	100.00	100.00	100.00	0	86.00	96.00	100.00	100.00	77.00	100.00	100.00	100.00
	24 h		97.00	99.00	97.00		100.00	100.00	100.00	1		85.00	94.00	74.00		60.00	100.00	100.00
	48 h		95.00	100.00	100.00		100.00	95.00	90.00	2		81.00	94.00	81.00		71.00	87.00	71.00
	72 h		100.00	99.00	97.00		83.00	100.00	100.00	3		77.00	96.00	81.00		100.00	85.00	40.00
										4		91.00	99.00	95.00		67.00	100.00	100.00
9	0 h	96.00	100.00	99.00	97.00	0.00	0.00	0.00	0.00	0	88.00	100.00	100.00	100.00	77.00	33.00	100.00	100.00
	24 h		97.00	98.00	94.00		0.00	0.00	0.00	1		88.00	96.00	82.00		100.00	91.00	67.00
	48 h		89.00	100.00	100.00		0.00	0.00	0.00	2		79.00	96.00	83.00		100.00	89.00	80.00
	72 h		100.00	98.00	94.00		0.00	0.00	0.00	3		79.00	97.00	90.00		75.00	89.00	75.00
										4		100.00	96.00	82.00		0.00	100.00	0.00

**Table 6:** Early Infection Detection and Severity Grading of Potato Bacterial Wilt Using Characteristic Bands Extracted by CARS and the PCA-LDA Model.

Pre-Processing Technique	Hours Post Inoculation	CV Detection of Training Data Set (%)				Test Data Set (%)				Disease Score	CV Detection of Training Data Set (%)				Test Data Set (%)			
		Accuracy	Sensitivity	Specificity	Precision	Accuracy	Sensitivity	Specificity	Precision		Accuracy	Sensitivity	Specificity	Precision	Accuracy	Sensitivity	Specificity	Precision
1	0 h	79.00	88.00	97.00	90.00	82.00	90.00	97.00	90.00	0	66.00	72.00	94.00	74.00	68.00	40.00	95.00	67.00
	24 h		77.00	93.00	79.00		70.00	97.00	88.00	1		49.00	91.00	56.00		60.00	82.00	46.00
	48 h		72.00	88.00	69.00		100.00	83.00	67.00	2		54.00	91.00	60.00		90.00	90.00	69.00
	72 h		81.00	94.00	81.00		70.00	100.00	100.00	3		79.00	88.00	63.00		70.00	93.00	70.00
										4		77.00	94.00	77.00		80.00	100.00	100.00
2	0 h	83.00	100.00	97.00	91.00		100.00	100.00	100.00	0	76.00	81.00	98.00	91.00	72.00	70.00	100.00	100.00
	24 h		85.00	94.00	85.00		80.00	93.00	80.00	1		68.00	93.00	69.00		70.00	90.00	64.00
	48 h		70.00	91.00	74.00	82.00	70.00	87.00	64.00	2		67.00	93.00	72.00		80.00	90.00	67.00
	72 h		81.00	95.00	83.00		80.00	97.00	89.00	3		76.00	89.00	64.00		60.00	90.00	60.00
										4		90.00	97.00	88.00		80.00	95.00	80.00
3	0 h	80.00	94.00	97.00	91.00	25.00	100.00	0.00	25.00	0	72.00	81.00	95.00	81.00	52.00	100.00	75.00	50.00
	24 h		85.00	92.00	79.00		0.00	100.00	0.00	1		57.00	92.00	64.00		20.00	70.00	14.00
	48 h		68.00	90.00	71.00		0.00	100.00	0.00	2		72.00	90.00	65.00		70.00	95.00	78.00
	72 h		75.00	94.00	79.00		0.00	100.00	0.00	3		71.00	90.00	64.00		0.00	100.00	0.00
										4		79.00	97.00	89.00		70.00	100.00	100.00
4	0 h	95.00	100.00	99.00	97.00	90.00	90.00	100.00	100.00	0	84.00	89.00	97.00	86.00	76.00	80.00	100.00	100.00
	24 h		95.00	99.00	97.00		100.00	97.00	91.00	1		73.00	95.00	77.00		100.00	90.00	71.00
	48 h		88.00	98.00	95.00		100.00	90.00	77.00	2		87.00	95.00	81.00		80.00	85.00	57.00
	72 h		97.00	96.00	90.00		70.00	100.00	100.00	3		82.00	93.00	76.00		50.00	100.00	100.00
										4		87.00	100.00	100.00		70.00	95.00	78.00
5	0 h	93.00	94.00	98.00	94.00	25.00	0.00	100.00	0.00	0	91.00	97.00	100.00	100.00	44.00	10.00	100.00	100.00
	24 h		95.00	97.00	93.00		0.00	100.00	0.00	1		84.00	98.00	91.00		100.00	63.00	40.00
	48 h		93.00	97.00	93.00		0.00	100.00	0.00	2		90.00	97.00	90.00		0.00	95.00	0.00
	72 h		92.00	98.00	94.00		100.00	0.00	25.00	3		92.00	93.00	78.00		10.00	100.00	100.00
										4		92.00	100.00	100.00		100.00	72.00	48.00
6	0 h	91.00	97.00	99.00	97.00	85.00	100.00	100.00	100.00	0	89.00	94.00	99.00	97.00	84.00	100.00	100.00	100.00
	24 h		97.00	98.00	95.00		80.00	100.00	100.00	1		86.00	97.00	89.00		100.00	97.00	91.00
	48 h		85.00	95.00	87.00		90.00	83.00	64.00	2		92.00	94.00	80.00		80.00	88.00	62.00
	72 h		86.00	95.00	86.00		70.00	97.00	88.00	3		87.00	95.00	82.00		70.00	100.00	100.00
										4		85.00	100.00	100.00		70.00	95.00	78.00
7	0 h	97.00	97.00	100.00	100.00	88.00	100.00	100.00	100.00	0	87.00	97.00	98.00	92.00	88.00	100.00	100.00	100.00
	24 h		100.00	99.00	97.00		80.00	93.00	80.00	1		78.00	97.00	88.00		100.00	95.00	83.00
	48 h		97.00	97.00	93.00		80.00	90.00	73.00	2		90.00	95.00	81.00		90.00	93.00	75.00
	72 h		92.00	99.00	97.00		90.00	100.00	100.00	3		82.00	94.00	78.00		60.00	100.00	100.00
										4		90.00	100.00	100.00		90.00	97.00	90.00
8	0 h	89.00	97.00	98.00	94.00	90.00	90.00	100.00	100.00	0	89.00	97.00	98.00	92.00	84.00	90.00	97.00	90.00
	24 h		95.00	98.00	95.00		90.00	93.00	82.00	1		76.00	98.00	90.00		100.00	95.00	83.00
	48 h		85.00	93.00	83.00		100.00	93.00	83.00	2		92.00	97.00	88.00		90.00	90.00	69.00
	72 h		81.00	95.00	85.00		80.00	100.00	100.00	3		87.00	94.00	79.00		60.00	100.00	100.00
										4		95.00	100.00	100.00		80.00	97.00	89.00
9	0 h	93.00	100.00	98.00	94.00	53.00	20.00	100.00	100.00	0	88.00	97.00	97.00	90.00	72.00	40.00	100.00	100.00
	24 h		95.00	99.00	97.00		100.00	63.00	48.00	1		81.00	98.00	91.00		90.00	80.00	53.00
	48 h		93.00	95.00	88.00		90.00	73.00	53.00	2		90.00	95.00	81.00		90.00	90.00	69.00
	72 h		86.00	98.00	94.00		0.00	100.00	0.00	3		87.00	96.00	85.00		60.00	97.00	86.00
										4		87.00	99.00	97.00		80.00	97.00	89.00

## Data Availability

The data presented in this study are available on request from the corresponding author.

## Abbreviations

The following abbreviations are used in this manuscript:

HSI	Hyperspectral Imaging
PLS-DA	Partial Least Squares-Discriminant Analysis
PCA-LDA	Principal Component Analysis-Linear Discriminant Analysis
CARS	Competitive Adaptive Reweighted Sampling
SNV	Standard Normal Variate
MSC	Multiplicative Scatter Correction
SG	Savitzky-Golay
MC	Mean Centering
NOR	Normalization
NIR	Near-Infrared
ROI	Region of Interest
PCR	Polymerase Chain Reaction
ELISA	Enzyme-Linked Immunosorbent Assay
UAV	Unmanned Aerial Vehicle
